# Evaluation of the killing effects of UV_254_ light on common airborne porcine viruses

**DOI:** 10.3389/fvets.2025.1512387

**Published:** 2025-01-31

**Authors:** YingWu Qiu, QunHui Li, WenKai Zhao, Hao Chang, JunHua Wang, Qi Gao, Qingfeng Zhou, GuiHong Zhang, Lang Gong, LianXiang Wang

**Affiliations:** ^1^Guangdong Provincial Key Laboratory of Zoonosis Prevention and Control, College of Veterinary Medicine, South China Agricultural University, Guangzhou, China; ^2^Guangdong Provincial Key Laboratory of Livestock and Poultry Health and Environmental Control, Yunfu, China; ^3^Foshan Comwin Light & Electricity Co., Ltd., Foshan, China; ^4^African Swine Fever Regional Laboratory of China (Guangzhou), Guangzhou, China

**Keywords:** UV radiation, air disinfection, ASFV, PRRSV, PEDV

## Abstract

UV exposure is a common method of disinfection and sterilization. In the present study, the parallel beam test was performed to collect fluids containing infectious viruses using a parallel beam apparatus after UV_254_ irradiation (0, 0.5, 1, 3, 5, 7, 10, or 20 mJ/cm^2^). The air sterilization test was performed by irradiating the air in the ducts with UV_254_ light (0, 1, 2, 3, 4, or 6 mJ/cm^2^) to collect airborne particles containing viruses through the air sterilization equipment. Furthermore, viral inactivation was assessed based on cytopathic effect (CPE) detection and immunofluorescent assays (IFA). Both the CPE and immunofluorescence signal intensity decreased as the UV_254_ dose increased. The UV_254_ doses required to inactivate ASFV (10^7.75^ copies/mL), PRRSV (10^6.29^ copies/mL), and PEDV (10^7.71^ copies/mL) in the water were 3, 1, and 1 mJ/cm^2^, respectively. The UV_254_ dose required to inactivate ASFV (10^4.06^ copies/mL), PRRSV (10^3.06^ copies/mL), and PEDV (10^4.68^ copies/mL) in the air was 1 mJ/cm^2^. This study provides data required for biosecurity prevention and control in swine farms.

## 1 Introduction

China is the world’s largest producer and consumer of pork, producing approximately 53% of the global pork supply (1). Furthermore, pork is the main source of high-quality protein for Chinese residents, with the consumption accounting for 62% of total meat consumption (2). Infectious diseases represent a major constraint to pig production (3). Since the first outbreak of African swine fever (ASF) in China in August 2018, ASF, porcine reproductive and respiratory syndrome (PRRS), and porcine epidemic diarrhea (PED) have emerged as the three most serious viral diseases in Chinese pig farms (4). These diseases are highly transmissible and pathogenic, with rapid mutation of the virulent strains, resulting in abortions in sows, growth delay in fattening pigs, and mass mortality among piglets (5, 6). When these diseases occur on pig farms, it is difficult to achieve decontamination because of the labor and resources required to control the spread of the disease in the herd. Notably, ASF virus (ASFV), PRRS virus (PRRSV), and PED virus (PEDV) can be transmitted through the air, further complicating disease prevention and control efforts in the entire Chinese pig farming industry (7–10).

UV disinfection is one of the most commonly used methods for preventing air-mediated microbial disease transmission because of its low cost, simple installation, ease of maintenance, and significant effectiveness (11, 12). UV light can inactivate pathogenic microorganisms through several mechanisms, such as the formation of cyclobutane pyrimidine dimers in nucleic acids, which ultimately inhibit transcription and replication (13). In addition, the generation of reactive oxygen species (ROS) results in the oxidation of macromolecules such as lipids, proteins, and carbohydrates inside the cells and leads to cell membrane and cell wall damage (14). Table 1 provides a summary of recent studies on the effectiveness of UV in inactivating various viruses. From these references, we can identify that in addition to the UV dose, important factors affecting UV disinfection include the wavelength of the UV light used, the type of virus, the environmental conditions, and the medium through which UV light is transmitted.

**Table 1:** Killing effect of ultraviolet light on viruses.

Virus type	Killing dose	Virus counting (viability) methods	Ultraviolet length	Inactivation rate constant	Medium	Article
Fr bacteriophage	0.5 J/cm^2^ 99.99 percent reduction	Plaque infectivity test	405		Viral fluid	(18)
ΦX174 bacteriophage	5 J/cm^2^ 90 percent reduction					
MS2 bacteriophage	679 J/cm^2^ 99.68 percent reduction	Plaque infectivity test	365–375		Viral fluid	(19)
PhiX-174 bacteriophage	16.1 mJ/cm^2^ 99.97–99.99 percent reduction	Plaque infectivity test	280		Viral fluid	(20)
MS2 bacteriophage	16.1 mJ/cm^2^ 99.97–99.99 percent reduction					
MS2 bacteriophage	143.4 mJ/cm^2^ 99.99–99.9996 percent reduction					
SARS-CoV-2	1.25 mJ/cm^2^ 90 percent reduction	TCID_50_	254	0.79	Water	(30)
	0.6 mJ/cm^2^ 90 percent reduction	TCID_50_	220	1.5		
Adenovirus	10 mJ/cm^2^ 99.99 percent reduction	qPCR and Plaque infectivity test	210		Water	(51)
	10 mJ/cm^2^ 99.9 percent reduction		220			
H1N1 influenza virus	10 mJ/cm^2^ 99.99 percent reduction	IFA	207–222	1.8	Air	(52)
SARS-CoV-2	10 mJ/cm^2^ 99.99 percent reduction	IFA	254		Air	(53)
SARS-CoV-2	4 mJ/cm^2^ inactivation 99.999%	TCID_50_	222	12.4	Air	(29)
SARS-CoV-2	2 mJ/cm^2^ 99.9 percent inactivation	TCID_50_/IFA	222	4.1	Air	(54)
SARS-CoV-2	1,048 mJ/cm^2^ inactivation 99.999 percent	TCID_50_	254		Viral fluid	(55)
SARS-CoV-2	10.25 to 23.71 mJ/cm^2^ inactivation 99.99 percent	TCID_50_	254		Stainless steel, plastic and glass	(56)
SARS-CoV-2	3.7 mJ/cm^2^ inactivates 99.9 percent	qPCR	254		Water	(57)
SARS-CoV-2	15 mJ/cm^2^ to inactivate 105 TCID_50_ virus solution	TCID_50_	253.7		Viral fluid	(58)
SARS-CoV-2	0.28 mJ/cm^2^ 99.2 percent inactivation	qPCR	254		Air	(59)
SARS-CoV-2	10 mJ/cm_2_ inactivation	TCID_50_/IFA	222/230		Water and saliva	(60)
SARS-CoV-2	15 mJ/cm^2^ 99.99 percent inactivation	TCID_50_	222		Viral fluid	(61)
SARS-CoV-2	7.4 mJ/cm^2^ inactivation	TCID_50_	254		—	(62)
SARS-CoV-2	3.6 mJ/cm^2^ inactivation	Plaque infectivity test	254		Viral fluid	(63)
SARS-CoV-2	3.5 mJ/cm^2^ inactivation	IFA	254		Viral fluid	(64)

Previous studies have shown that UV disinfection is an effective method to inactivate a wide range of pathogenic microorganisms, including various phages and viruses such as SARS-CoV-2 (15–22). This study aimed to evaluate the inactivating effect of UV_254_ light, a UV-C wavelength, on common airborne porcine viruses, providing critical data for the prevention and control of animal diseases.

## 2 Materials and methods

### 2.1 Viruses and cells

ASFV, PRRSV, and PEDV were obtained from the National Regional Laboratory for African Swine Fever (Guangzhou) of South China Agricultural University (Guangzhou, China). Porcine primary alveolar macrophages (PAMs) were isolated from the bronchoalveolar lavage fluid of 4-week-old healthy piglets. Marc-145 and Vero cells were obtained via direct passage. Then, 1% porcine erythrocyte suspension was prepared using EDTA-treated fresh porcine blood. Viral stock solutions were diluted to 1 × 10^6^ and 1 × 10^3^ TCID_50_ using autoclaved ddH_2_O for parallel beam UV_254_ experiments. A nebulizer aerosolized 15 mL of virus stock solution for each air sampler operation, with a collection duration of 15 min per sampling. Three replications of each experiment were performed. All viral manipulations in cells were conducted at the BSL-3 laboratory of the College of Veterinary Medicine, South China Agricultural University.

### 2.2 Parallel beam UV experiment

**Figure 1:**
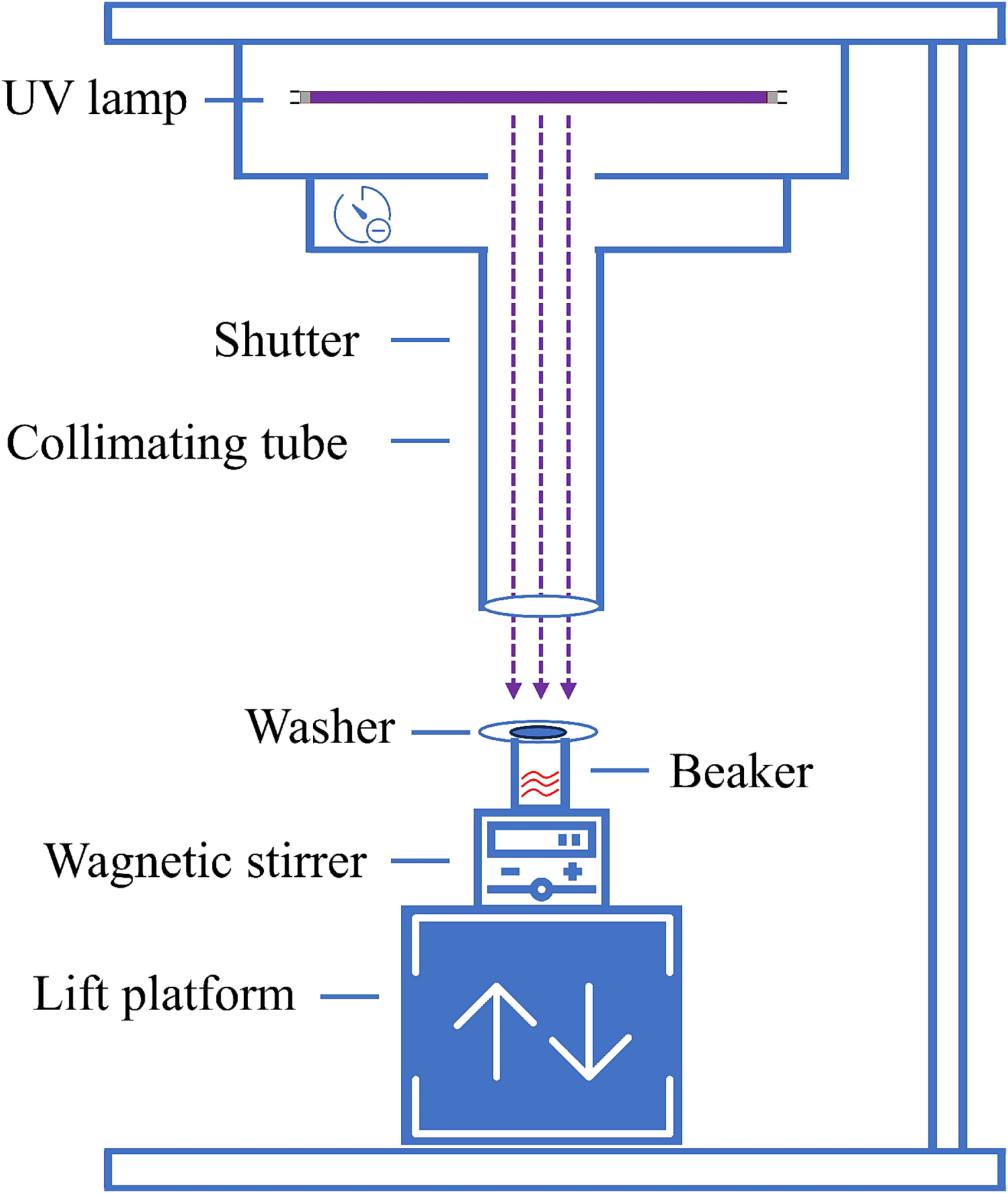
Parallel beam UV meter. The parallel beam apparatus, designed for precise UV_254_ experiments, comprises UV_254_ lamp, shutter, collimator tube, washer, beaker, magnetic stirrer, and lifter (23).

As shown in Figure 1, compared with traditional UV radiometers, the parallel beam apparatus optimizes beam collimation and uniformity, enabling more precise control and measurement of UV_254_ irradiance, thereby enhancing the reliability of experimental results (23). Parallel beam UV_254_ experiments were performed by fixing the UV_254_ illumination of the light source and using different TCID_50_ values for viruses and varying durations of UV_254_ irradiation. As presented in Supplementary Table S1, the duration of irradiation using the 36-W UV_254_ lamp (wavelength = 254 nm) were set to 0, 3.5, 6.9, 20.8, 34.6, 48.4, 69.2, or 138.4 s, and the UV_254_ dose was set to 0, 0.5, 1, 3, 5, 7, 10, or 20 mJ/cm^2^. After irradiating ASFV (TCID_50_ = 1 × 10^6^/CT = 16.45, TCID_50_ = 1 × 10^3^/CT = 29.64), PRRSV (TCID_50_ = 1 × 10^6^/CT = 14.36, TCID_50_ = 1 × 10^3^/CT = 25.36), and PEDV (TCID_50_ = 1 × 10^6^/CT = 16.60, TCID_50_ = 1 × 10^3^/CT = 27.47), viral inactivation was detected by assessing cytopathic effects (CPEs) and performing IFAs to determine the UV_254_ dose required for killing effects. Three replications of each experiment were performed.

### 2.3 Air sterilization experiment

**Figure 2:**
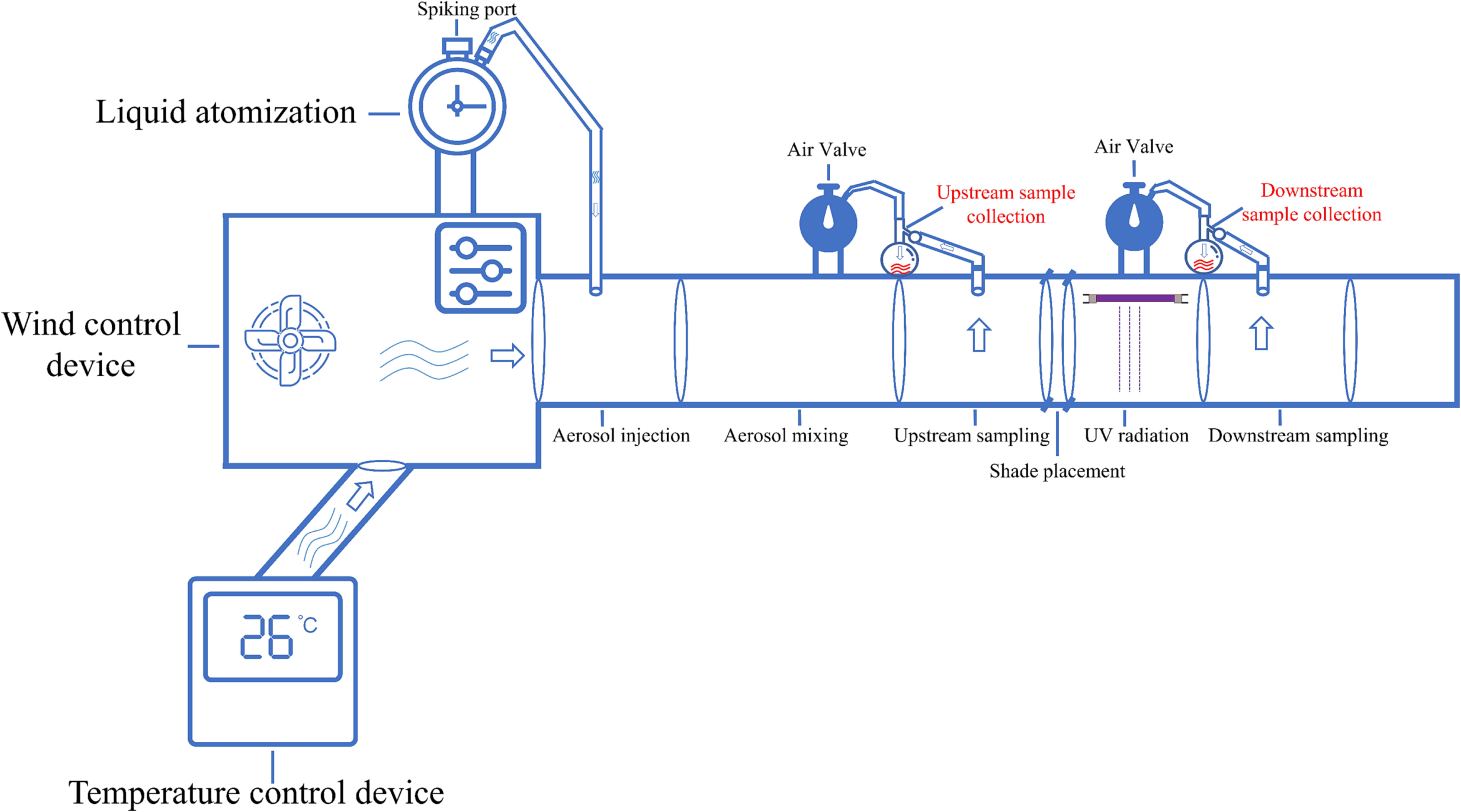
Equipment for air sterilization in a duct. The air disinfection equipment contained a temperature regulation device, wind speed controller, nebulizer (with liquid gasification function), air sampler (with gas liquefaction function), UV_254_ device, and ventilation duct to simulate UV_254_ disinfection of the air (50).

**Figure 3:**
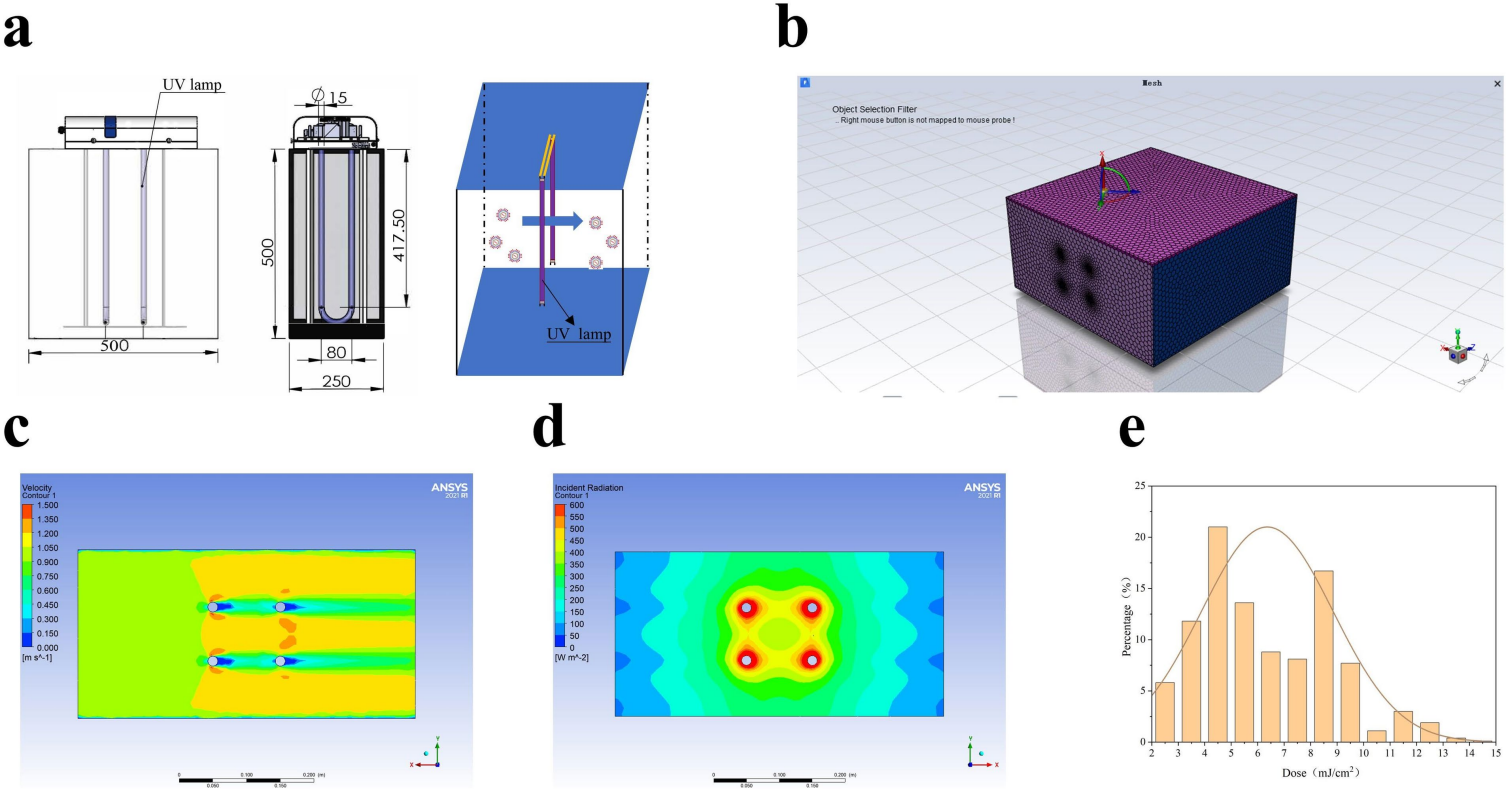
Duct calculation method. **(A)** UV_254_ sterilization equipment. The equipment included a closed pipeline disinfection chamber with a cross-section of 500 × 250 mm^2^ and a total length of 500 mm. Two built-in power sources (75 W each), and Kewei brand U-shaped low-pressure, high-intensity UV light (100 mm apart) with a UVC efficiency of 32% placed perpendicular to the wind direction. **(B)** Grid schematic. The structured grid shown in the figure was used to divide the sterilized area for simulation. A total of 288,738 grid cells were applied in the study. **(C)** Velocity field distribution. With an inlet wind speed of 1 m/s, the internal velocity field exhibited an axisymmetric distribution. Due to the bypassing effect of the lamps, the minimum velocity appeared in the downstream region of the light. However, the velocity variation across the flow field was minimal, resulting in a relatively uniform particle residence time in the range of 0.4–0.6 s. **(D)** Radiation intensity distribution. The distribution of internal radiation intensity indicated that the highest intensity occurred near the lamps, gradually decreasing along the radial direction from the light surface. **(E)** UV_254_ dose distribution. The radiation dose of particles flowing through the UV_254_ disinfection equipment is shown in figure. Based on the DPM model, 1,000 particles were injected simultaneously, and statistical analysis calculated the effective dose of the model as 6.086 mJ/cm^2^.

As shown in Figure 2, the air disinfection experiment was performed by adjusting the UV_254_ illumination intensity and wind speed over a fixed UV_254_ irradiation time. The CT values of ASFV, PRRSV, and PEDV stock solutions were 13.5, 12.36, and 11.01, respectively. As illustrated in Supplementary Table S2, the temperature was set to 26°C. Meanwhile, the power of the UV_254_ light (wavelength = 254 nm) was set to 0, 50, or 150 W; the airflow rates in the air sampler and wind tunnel were set to 1 m/s and 2 m/s, respectively, based on the required UV dose. As shown in Figure 3, the corresponding UV_254_ dose was set to 0, 1, 2, 3, 4, or 6 mJ/cm^2^ based on the simulation. First, the air sampler was used to collect airborne particles containing viruses upstream of the sampling section 30 s after nebulization. Subsequently, similar particles were collected downstream. Each collection lasted 15 min to ensure sufficient capture of airborne particles containing viruses. Note that the air sampler must be replaced after each collection, and the downstream sampler should not be connected while the upstream sampler is in operation. The air collected before and after UV_254_ irradiation was dissolved into the culture medium, and viral inactivation was determined by assessing CPEs and performing IFAs. The end of the ventilation duct was equipped with an exhaust gas treatment unit to inhibit the release of viruses into the environment. Three replications of each experiment were performed.

### 2.4 Nucleic acid extraction and quantitative qPCR

After treatment, nucleic acids were extracted from ASFV, PRRSV, and PEDV using RaPure Viral RNA/DNA Kit (Guangzhou, China) as per the manufacturer’s instructions, and qPCR was performed using the reaction system and procedure described previously (24–26). Three assays were performed for each sample. Regarding the results, negative samples had no CT values, positive samples had CT values of ≤34.0 with typical amplification curves, and suspicious samples had CT values of >34.0 with typical amplification curves. If two samples were considered suspicious, the result of the third sample was used.

### 2.5 Parameters of the parallel beam UV_254_ meter

The impact of UV_254_ light on pathogenic microorganisms is determined by the UV_254_ dose they receive. The UV_254_ is defined as (27):

$$\mathrm{Dose}=\overset{t}{\underset{0}{\int }}Idt$$

where UV_254_ dose is measured in mJ/cm^2^, *I* represents the UV_254_ light intensity received by the microorganism at a point on its trajectory (mW/cm^2^), and *t* is the irradiation time (s). The average UV_254_ intensity received by microorganisms in the water is defined as (28):

$$E_{ave}=0.98\left[\frac{E_{0}}{L}\left(\frac{{\left(T\right)}^{L}-1}{\ln\left[T\right]}\right)\right]$$

where *E*_ave_ represents the average illuminance in the water (mW/cm^2^), *E*_0_ represents the incident irradiance (mW/cm^2^), *L* is the depth of the solution irradiated by the collimated beam (cm), *A* is the UV_254_ absorbance at a 1-cm light range, and *T* = 1 − *A*. Considering all irradiated pathogenic microorganisms as a collective group, the total UV_254_ dose received can be calculated as: $\mathrm{Dose}=E_{ave}\times t$ (28).

### 2.6 Air sterilization parameters

The UV_254_ radiation dose received by a pathogenic microorganism in the reactor is determined by its path and exposure time. The relationship between microbial inactivation efficiency and UV_254_ dose is defined as (29):

$$-\mathrm{lg}\left(\frac{N}{N_{0}}\right)=A\times F+B$$

where *F* is the UV_254_ dose (mJ/cm^2^); *N*_0_ and *N* represent the microbial content before and after irradiation, respectively; and *A* and *B* are the disinfection kinetic parameters measured using a parallel beam meter. By determining the UV_254_ dose received by each microcluster at the reactor’s exit, the corresponding inactivation rate can be calculated. The overall inactivation rate is the combined effect of all microclusters (29):

$${\left(\frac{N}{N_{0}}\right)}_{\mathrm{total}}=\frac{\sum 10^{-\left(A\times F_{i}+B\right)}}{T}$$

where *F_i_* represents the UV_254_ dose received by each microcluster at the exit (mJ/cm^2^) and *T* is the total number of microclusters. From this, the total effective dose (RED) is defined as (29):

$$\mathrm{RED}=-\frac{\left(\mathrm{lg}{\left(\frac{N}{N_{0}}\right)}_{\mathrm{total}}+B\right)}{A}$$

### 2.7 Determination of virus infectivity

ASFV samples treated with different UV_254_ doses were used to infect PAMs. Similarly, treated PRRSV samples were used to infect Marc-145 cells, and treated PEDV samples were used to infect Vero cells. Virus infectivity was determined by assessing CPEs and performing IFAs. In brief, PAMs, Marc-145 cells, and Vero cells were inoculated into 96-well plates, and viral suspensions (ASFV diluted in RPMI-1640 containing 10% FBS, PRRSV diluted in Dulbecco’s modified Eagle medium [DMEM] containing 2% FBS, and PEDV diluted in DMEM containing 7 μg/mL trypsin) were added to the plates at a 10-fold gradient (1 × 10^−1^ to 1 × 10^−10^), with columns 1 and 12 serving as controls. Viral infectivity was confirmed via the IFA using antibodies specific for ASFV, PRRSV, and PEDV, and the TCID_50_ was determined using the Reed and Muench method.

### 2.8 *In vitro* biological characterization of viruses after irradiation

PAMs, Marc-145 cells, and Vero cells were infected with ASFV, PRRSV, and PEDV, respectively, following UV irradiation, and viral infectivity was confirmed by assessing CPEs and performing IFAs. In brief, PAMs, Marc-145 cells, and Vero cells were inoculated into 96-well plates, and viral suspensions were added to the plates at a 10-fold gradient (1 × 10^−1^ to 1 × 10^−10^), with columns 1 and 12 serving as controls. Three replications of each experiment were performed. Viral fluids were collected at 6-h intervals to construct *in vitro* growth curves using GraphPad Prism 8 software (GraphPad, San Diego, CA, United States).

### 2.9 Data analysis

The UV_254_ dose responses based on UVC at 254 nm were evaluated using a pseudo first-order inactivation kinetics model in the log_10_ scale as follows (30):

$$\log_{10}\;I=\log_{10}\left(\frac{N_{0}}{N}\right)=k\times D$$

where log_10_ *I* represents the reduction in infectivity on the log_10_ scale; *N*_0_ and *N* represent the infectivity of virus samples before and after UV_254_ exposure, respectively; *D* represents the UV fluence in mJ/cm^2^; and *k* represents the pseudo first-order inactivation rate constant in cm^2^/mJ computed using a log_10_-scale kinetic model. The log_10_ scale inactivation rate constant was used, which facilitated the calculation of log inactivation using the rate constant.

## 3 Results

### 3.1 Viral nucleic acids were not degraded by UV_254_ irradiation at different doses

**Figure 4:**
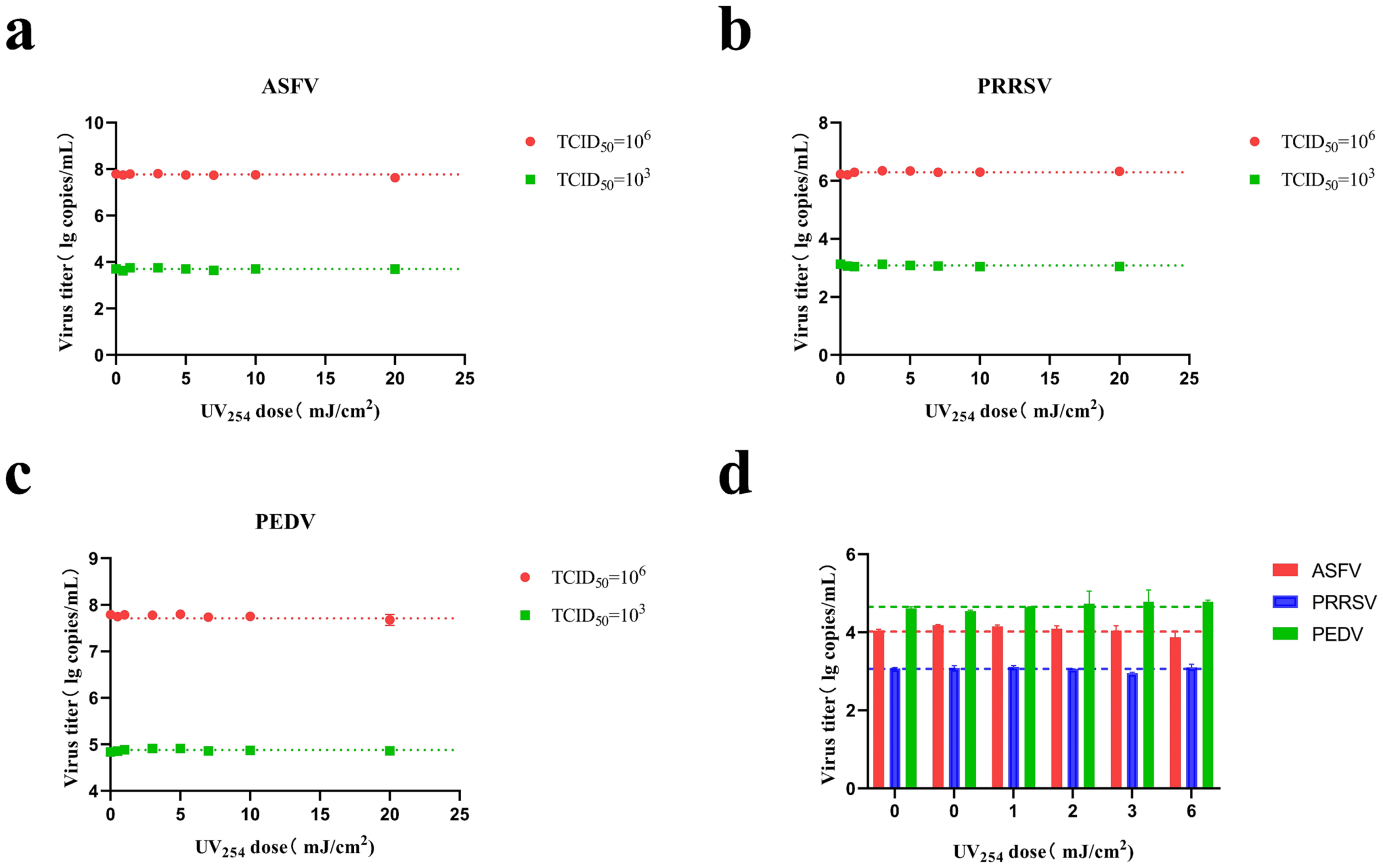
Changes in the CT values of ASFV, PRRSV, and PEDV after irradiation with different UV doses. **(A)** Irradiation of ASFV solution (TCID_50_ = 1 × 10^3^ and 1 × 10^6^) using a parallel beam UV device. **(B)** Irradiation of PRRSV solution (TCID_50_ = 1 × 10^3^ and 1 × 10^6^) using a parallel beam UV device. **(C)** Irradiation of PEDV solution (TCID_50_ = 1 × 10^3^ and 1 × 10^6^) using a parallel beam UV device. **(D)** Irradiation of aerosolized ASFV, PRRSV, and PEDV in air disinfection ducts.

The ASFV, PRRSV, and PEDV solutions were irradiated with different UV_254_ doses (0, 0.5, 1, 3, 5, 7, 10, and 20 mJ/cm^2^), as presented in Figures 4A–C. The copy numbers of ASFV, PRRSV, and PEDV did not differ significantly among the treatment groups. Further, ASFV, PRRSV, and PEDV were nebulized and then irradiated with different UV_254_ doses (0, 1, 2, 3, and 6 mJ/cm^2^). As shown in Figure 4D, the copy numbers of the viruses were not altered by nebulization. This suggests that low-dose UV_254_ irradiation does not lead to significant nucleic acid degradation in ASFV, PRRSV, and PEDV.

### 3.2 Low-dose UV exposure reduces the abundance of infectious virus in the samples

**Figure 5:**
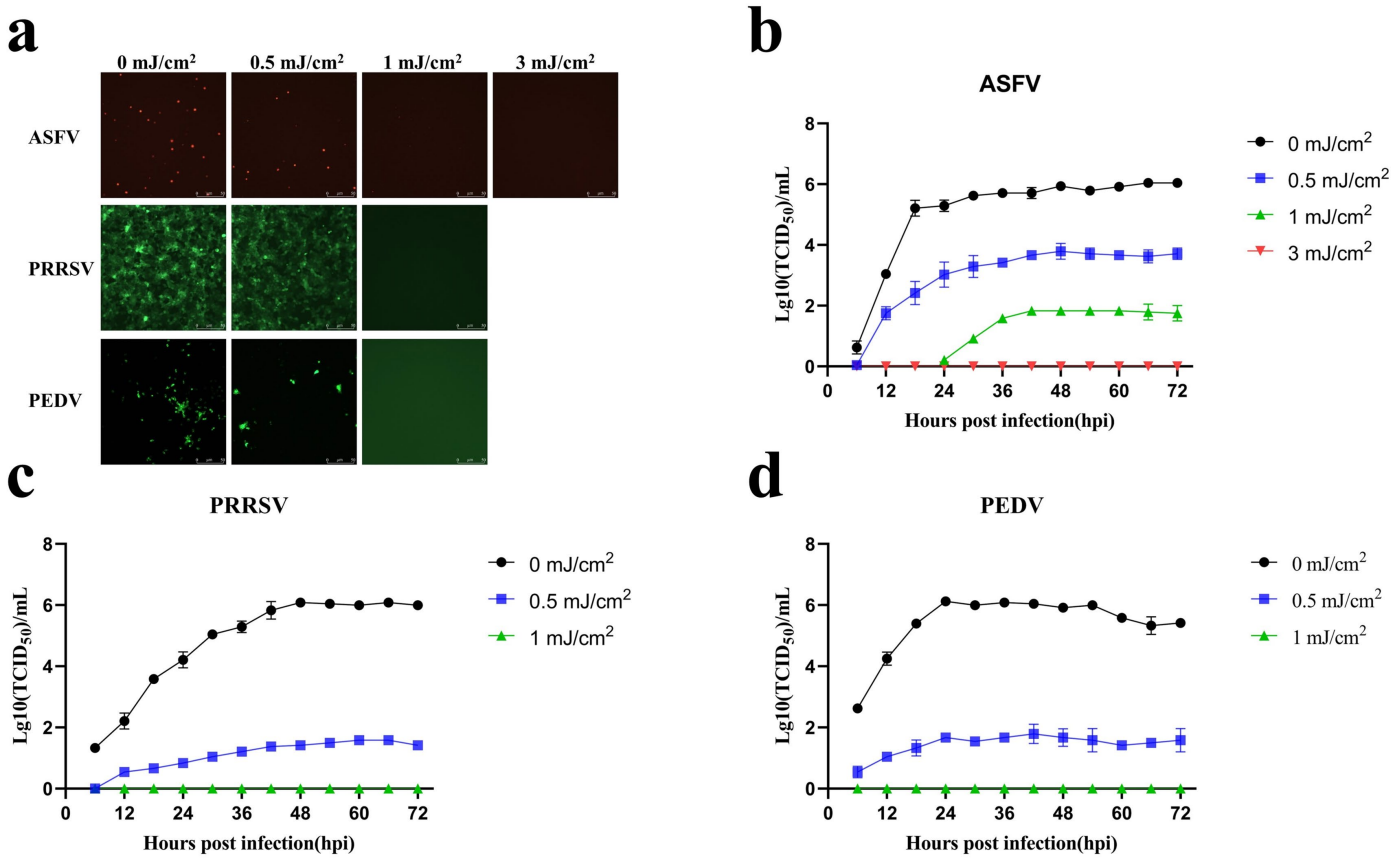
Low-dose UV_254_ irradiation reduces the abundance of infectious virus in the samples. **(A)** Changes in the fluorescence signals of ASFV, PRRSV, and PEDV after treatment with different UV doses. **(B)** Growth curves of ASFV after treatment with different UV_254_ doses. **(C)** Growth curves of PRRSV after treatment with different UV_254_ doses. **(D)** Growth curves of PEDV after treatment with different UV_254_ doses.

ASFV, PRRSV, and PEDV (TCID_50_ = 1 × 10^6^) were irradiated at different UV_254_ doses (0, 0.5, 1, 3, 5, 7, 10, and 20 mJ/cm^2^) and used to infect PAMs, Marc-145 cells, and Vero cells, respectively. As presented in Figure 5A, the fluorescence intensity of ASFV treated with UV_254_ doses of 0.5 and 1 mJ/cm^2^ was significantly lower than that of untreated ASFV, and no fluorescence was observed for ASFV treated with an external UV_254_ dose of 3 mJ/cm^2^. The fluorescence intensity of PRRSV treated with a UV_254_ dose of 0.5 mJ/cm^2^ was significantly lower than that of untreated PRRSV, and no fluorescence was observed for PRRSV treated with an external UV_254_ dose of 1 mJ/cm^2^. The fluorescence intensity of PEDV treated with a UV_254_ dose of 0.5 mJ/cm^2^ was significantly lower than that of untreated PEDV, and no fluorescence was observed for PEDV treated with an external UV_254_ dose of 1 mJ/cm^2^. As shown in Figures 5B–D, the infectivity of the viruses decreased significantly with increasing UV_254_ doses, and ASFV was more resistant to UV_254_ irradiation than PRRSV and PEDV. These results indicated that low-dose UV_254_ irradiation can reduce the infectivity of viruses in cells.

### 3.3 Quantification of UV_254_-induced inactivation of ASFV, PRRSV, and PEDV

**Figure 6:**
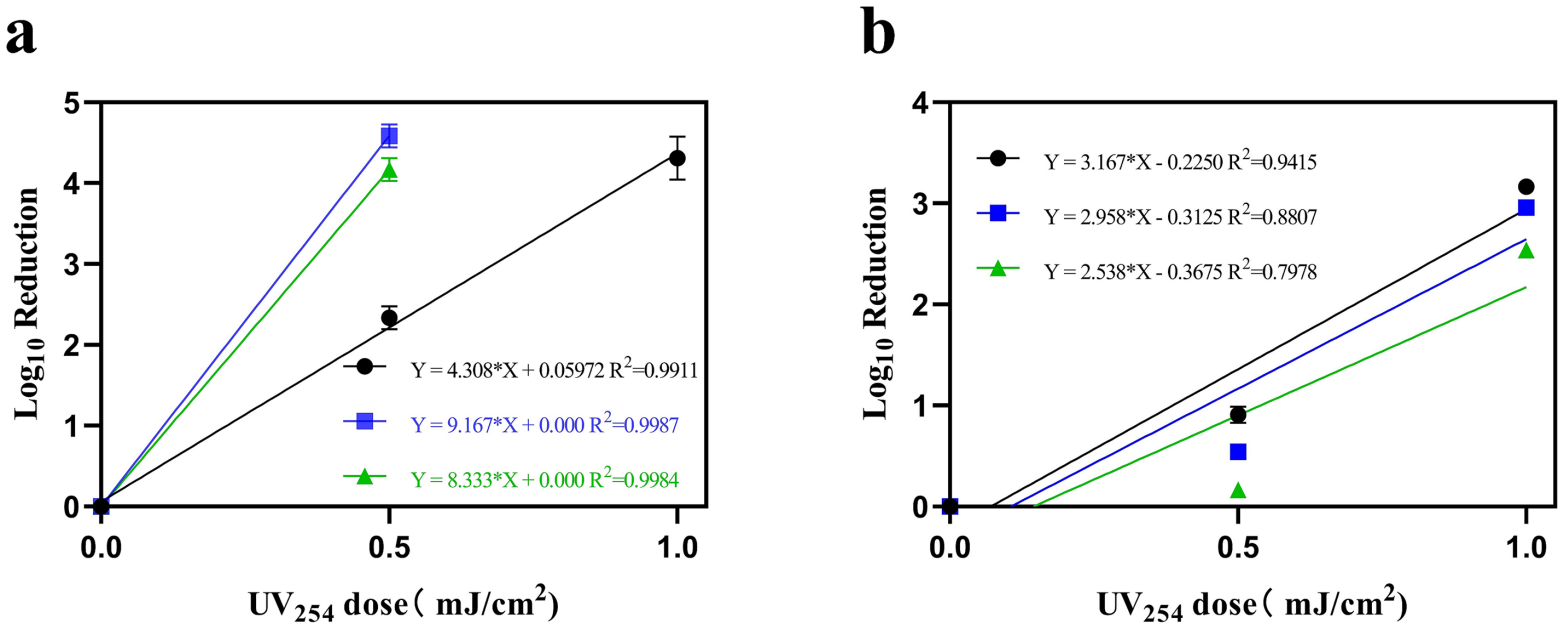
The relationship between the inactivation of ASFV, PRRSV, and PEDV in water **(A)** and air **(B)** with UV_254_ dose, measured by TCID_50_ relative to untreated virus controls. (Black indicates ASFV; blue indicates PRRSV; and green indicates PEDV).

Water and air containing ASFV, PRRSV, and PEDV were irradiated with different doses of UV_254_ and were subsequently used to infect PAMs, Marc-145 cells, and Vero cells, respectively. Figure 6A, linear regression analysis revealed a rate constant of 4.308 cm^2^/mJ (95% confidence interval = 3.943–4.674) for ASFV, which corresponds to a 90% inactivation dose (D_90_) of 0.23 mJ/cm^2^. In addition, the rate constant for PRRSV was 9.167 cm^2^/mJ (95% confidence interval = 8.704–9.629), which corresponds to a D_90_ of 0.11 mJ/cm^2^. Further, the rate constant for PEDV was 8.333 cm^2^/mJ (95% confidence interval = 7.871–8.796), corresponding to a D_90_ of 0.12 mJ/cm^2^. Figure 6B, linear regression analysis revealed a rate constant of 3.167 cm^2^/mJ (95% confidence interval = 2.461–3.872) for ASFV, which corresponds to a 90% inactivation dose (D_90_) of 0.32 mJ/cm^2^. In addition, the rate constant for PRRSV was 2.958cm^2^/mJ (95% confidence interval = 1.985–3.932), which corresponds to a D_90_ of 0.338 mJ/cm^2^. Further, the rate constant for PEDV was 2.538 cm^2^/mJ (95% confidence interval = 1.396–3.681), corresponding to a D_90_ of 0.394 mJ/cm^2^.

### 3.4 UV_254_ doses exceeding 1 mJ/cm^2^ inactivate ASFV, PRRSV, and PEDV in the air

**Figure 7:**
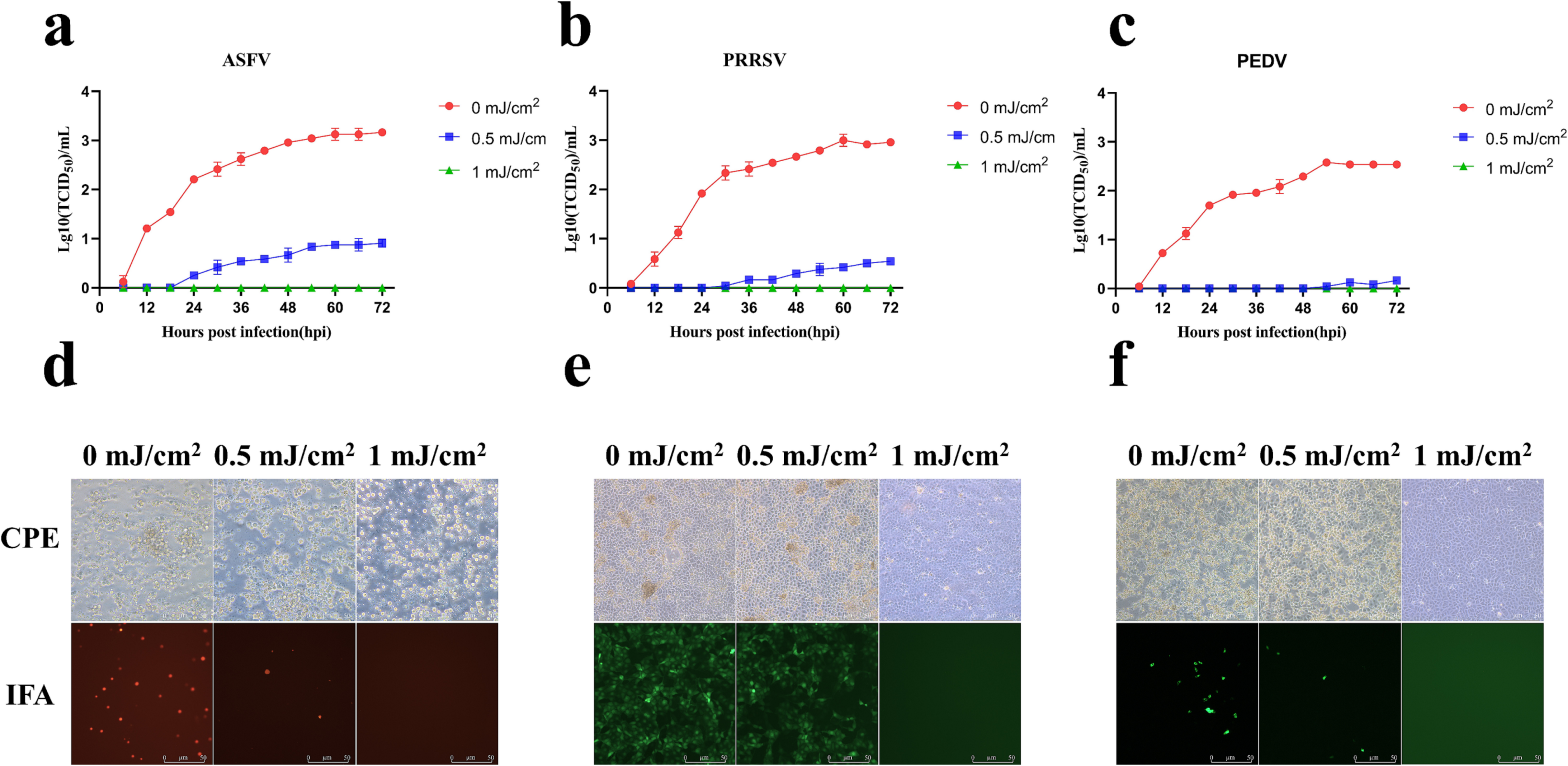
Replication of ASFV, PRRSV, and PEDV after UV_254_ treatment at a dose of 1 mJ/cm^2^. **(A–C)** Growth curves of ASFV, PRRSV, and PEDV. **(D–F)** CPEs and IFA data for ASFV, PRRSV, and PEDV.

ASFV, PRRSV, and PEDV were collected through an air sampler after irradiation with different UV_254_ doses (0, 1, 2, 3, and 6 mJ/cm^2^) and used to infect PAMs, Marc-145 cells, and Vero cells, respectively. As presented in Figure 7, ASFV, PRRSV, and PEDV irradiated with a UV_254_ dose of 1 mJ/cm^2^ lost the ability to infect cells, whereas untreated viruses caused obvious lesions in the cells within 48 h after inoculation. The IFA and growth curves indicated that the untreated viruses showed normal replication in the cells.

## 4 Discussion

The ASF outbreak in China in August 2018 led to major changes in pig farming patterns in China, including the introduction of biosecurity prevention and control (31, 32). Previous studies have revealed that the positivity rates of various swine diseases decreased significantly with the establishment of biosecurity prevention and control systems in Chinese pig farms (33). Disinfection is an important part of the biosafety system (34). Currently, chemical disinfection is commonly used in pig farms because of its ease of use and obvious inactivate effects against pathogenic microorganisms (35, 36). However, this disinfection method is associated with various problems, such as the presence of residual chemicals, secondary pollution, and formation of toxic disinfection by-products (DBPs). In addition, the types and usage of disinfectants applied on different objects are diverse, and some disinfectants are prone to cause damage to feed, food, and electronics. Therefore, chemical disinfection methods cannot be used in all scenarios in pig farms (37–42).

UV_254_ treatment is a physical disinfection method, and the use of the UVC band for UV_254_ irradiation leads to photochemical damage and ROS generation in pathogenic microorganisms, which affects the replication and transcription of genetic material and cause cell membrane and cell wall damage, ultimately leading to the death of microorganisms (13, 14, 27, 38, 43). Compared with chemical disinfection, UV_254_ disinfection is characterized by short disinfection time, high efficiency, broad germicidal spectrum, simple structure, small footprint, easy maintenance, and the absence of DBP production, resulting in its widespread use in multiple applications, such as air disinfection, water purification and wastewater treatment, food preservation, and medical applications (11, 12, 44, 45). The effectiveness of UV-mediated inactivation depends on the type of pathogenic microorganism and operating conditions, such as UV wavelength, UV intensity, and duration of irradiation. Moreover, environmental conditions can also affect the efficacy of UV-based inactivation (11, 46).

ASFV, PRRSV, and PEDV are the three most serious viral diseases that can be transmitted through the air to pig farms in China. Similar to SARS-CoV-2 in humans, these viruses can cause widespread and rapid damage in infected pigs if their spread is not controlled, as observed during the ASF outbreak in China in 2018 (31, 47–49). It is well known that UV_254_ treatment has a strong killing effect. Currently, although UV_254_ disinfection is widely used in pig farms, research on its killing effects on these three viruses is less extensive than that on SARS-CoV-2. Water and air are two important media for viral transmission. In the early stage of experimental designing, we reviewed a large number of studies on the killing effects of UV_254_ disinfection. We revealed that UV_254_ treatment has a stronger effect on viruses in the air than in viruses in the water. A UV_254_ dose of <1 mJ/cm^2^ can inactivate 99.9% of SARS-CoV-2 virions, and the killing effect of UV_254_ is stronger in pure water than in culture medium. Compared with other wavelengths, UV_254_ irradiation at a wavelength of 254 nm has a stronger killing effect (31, 47–49).

We investigated the UV_254_ dose required to inactivate ASFV, PRRSV, and PEDV in pure water using a UV_254_ parallel beam meter and then assessed its effects on viruses in the air using air sterilization equipment. We used primers and probes specific to ASFV-B646L, PRRSV-ORF6, and PEDV-M genes to detect the viral nucleic acid abundance of ASFV, PRRSV, and PEDV, respectively, before and after irradiation with different UV_254_ doses (parallel beam UV_254_ system: 0–20 mJ/cm^2^; air sterilization duct: 0–6 mJ/cm^2^). Further, we assessed viral infectivity by measuring CPEs and performing IFAs. The results revealed that low-dose UV_254_ irradiation did not significantly degrade viral nucleic acids or suppress viral infectivity. In addition, ASFV, PRRSV, and PEDV treated with UV_254_ doses of 3, 1, and 1 mJ/cm^2^, respectively, these viral fluids were found to be infectivity-incompetent. To more intuitively demonstrate the relationship of the UV_254_ dose with ASFV, PRRSV, and PEDV inactivation, the inactivation rate was quantified as the ratio of TCID_50_ before and after UV irradiation. ASFV was more resistant to UV_254_ irradiation than PRRSV and PEDV, probably because ASFV consists of a four-layered protein shell and an internal genome, which is apparently more complex in structure than the internal genomes of PRRSV and PEDV. The air sterilization experiment revealed good cell growth, no cell lesions, and no fluorescence in the 1 mJ/cm^2^ treatment group, suggesting that this dose is sufficient to inactivate ASFV, PRRSV, and PEDV. The stronger killing effects of UV_254_ in the air than in the water are likely attributable to the fact that UV_254_ can directly contact viruses in the air, whereas water refracts UV_254_ light. This experiment was performed under ideal conditions where in UV_254_ irradiation was applied directly to the viruses, resulting in killing effects at low doses. In real-word situations, the environment is intricate, and the number and size of dust particles in water and air can affect the efficiency of UV_254_ disinfection. Therefore, it may be necessary to increase the UV dose in practical applications. In summary, we believe that UV_254_ disinfection can be used in air filtration devices and other joint applications to detoxify air.

## 5 Conclusion

This study revealed that low-dose (0–20 mJ/cm^2^) UV_254_ irradiation significantly reduces viral infectivity without causing nucleic acid degradation. Using parallel beam UV_254_ apparatus, the UV_254_ doses required to inactivate ASFV, PRRSV, and PEDV were preliminarily determined to be 3, 1, and 1 mJ/cm^2^, respectively. The air disinfection experiment illustrated that a UV_254_ dose of 1 mJ/cm^2^ was sufficient to eradicate ASFV, PRRSV, and PEDV. These findings may provide a reference for the design and application of UV_254_ equipment in pig farms and lay a foundation for further research and development regarding viral disinfection.

## Data Availability

The original contributions presented in the study are included in the article/Supplementary material, further inquiries can be directed to the corresponding authors.
