# Supplementary material for: Evaluation of the killing effects of UV254 light on common airborne porcine viruses
Source: Front Vet Sci. 2025 Jan 31;12:1512387. doi: 10.3389/fvets.2025.1512387 (PMC11826351; doi:10.3389/fvets.2025.1512387)
Supplement: Supplementary file 1 [file Table_1.docx]

| **Supplementary Table 1.** Experimental conditions for UV parallel beam. | | | | | | | |
| --- | --- | --- | --- | --- | --- | --- | --- |
| **Virus** | **UV lamp power (W)** | **Illumination time (s)** | **UV intensity (mJ/cm^2^)** | **Virus titer (lg copies/mL)** | **Cellular infections** | **Virus titer (lg copies/mL)** | **Cellular infections** |
| ASFV | 36 | 0 | 0 | 7.79 | + | 3.71 | + |
|  |  | 3.5 | 0.5 | 7.74 | + | 3.63 | + |
|  |  | 6.9 | 1 | 7.78 | + | 3.75 | + |
|  |  | 20.8 | 3 | 7.81 | - | 3.75 | - |
|  |  | 34.6 | 5 | 7.75 | - | 3.71 | - |
|  |  | 48.4 | 7 | 7.74 | - | 3.65 | - |
|  |  | 69.2 | 10 | 7.75 | - | 3.70 | - |
|  |  | 138.4 | 20 | 7.63 | - | 3.69 | - |
| PRRSV | 36 | 0 | 0 | 6.23 | + | 3.13 | + |
|  |  | 3.5 | 0.5 | 6.20 | + | 3.07 | + |
|  |  | 6.9 | 1 | 6.29 | - | 3.05 | - |
|  |  | 20.8 | 3 | 6.35 | - | 3.13 | - |
|  |  | 34.6 | 5 | 6.34 | - | 3.08 | - |
|  |  | 48.4 | 7 | 6.29 | - | 3.06 | - |
|  |  | 69.2 | 10 | 6.30 | - | 3.04 | - |
|  |  | 138.4 | 20 | 6.32 | - | 3.05 | - |
| PEDV | 36 | 0 | 0 | 7.66 | + | 4.84 | + |
|  |  | 3.5 | 0.5 | 7.70 | + | 4.86 | + |
|  |  | 6.9 | 1 | 7.72 | - | 4.89 | - |
|  |  | 20.8 | 3 | 7.76 | - | 4.91 | - |
|  |  | 34.6 | 5 | 7.61 | - | 4.91 | - |
|  |  | 48.4 | 7 | 7.59 | - | 4.86 | - |
|  |  | 69.2 | 10 | 7.78 | - | 4.87 | - |
|  |  | 138.4 | 20 | 7.83 | - | 4.87 | - |

Note：“+” means CPE was observed, “-” means CPE was not observed.

| **Supplementary Table 2.** Air UV disinfection capacity. | | | | | | |
| --- | --- | --- | --- | --- | --- | --- |
| **Virus** | **UV lamp power (W)** | **Wind speed (m/s)** | **UV intensity (mJ/cm^2^)** | **Sampling segment** | **Virus titer (lg copies/mL)** | **Cellular infections** |
| ASFV | 0 | 2 | 0 | Upstream | 4.04 | + |
|  |  | 1 | 0 | Upstream | 4.17 | + |
|  | 50 | 2 | 1 | Downstream | 4.15 | - |
|  |  | 1 | 2 | Downstream | 4.09 | - |
|  | 150 | 2 | 3 | Downstream | 4.04 | - |
|  |  | 1 | 6 | Downstream | 3.87 | - |
| PRRSV | 0 | 2 | 0 | Upstream | 3.07 | + |
|  |  | 1 | 0 | Upstream | 3.08 | + |
|  | 50 | 2 | 1 | Downstream | 3.11 | - |
|  |  | 1 | 2 | Downstream | 3.06 | - |
|  | 150 | 2 | 3 | Downstream | 2.96 | - |
|  |  | 1 | 6 | Downstream | 3.10 | - |
| PEDV | 0 | 2 | 0 | Upstream | 4.61 | + |
|  |  | 1 | 0 | Upstream | 4.55 | + |
|  | 50 | 2 | 1 | Downstream | 4.64 | - |
|  |  | 1 | 2 | Downstream | 4.73 | - |
|  | 150 | 2 | 3 | Downstream | 4.78 | - |
|  |  | 1 | 6 | Downstream | 4.78 | - |

Note：“+” means CPE was observed, “-” means CPE was not observed.
